# Osteoporotic sacral insufficiency fracture: An easily neglected disease in elderly patients

**DOI:** 10.1097/MD.0000000000009100

**Published:** 2017-12-22

**Authors:** Feng-Chen Kao, Yao-Chun Hsu, Pao-Hsin Liu, Lee-Ren Yeh, Jung-Ting Wang, Yuan-Kun Tu

**Affiliations:** a Department of Orthopaedics, E-Da Hospital; b School of Medicine for International Students, I-Shou University, Kaohsiung; c School of Medicine, Big Data Research Center, Fu-Jen Catholic University; d Division of Gastroenterology, Fu-Jen Catholic University Hospital, New Taipei; e Graduate Institute of Clinical Medicine, China Medical University, Taichung; f Division of Gastroenterology and Hepatology, E-Da Hospital; g Department of Biomedical Engineering, I-Shou University; h Department of Radiology, E-Da Hospital, I-Shou University, Kaohsiung, Taiwan.

**Keywords:** osteoporotic compression fracture, sacral insufficiency fracture, sacroplasty

## Abstract

Sacral insufficiency fractures (SIFs) are easily neglected by clinical physicians.

The incidence of SIFs remains unclear in patients with symptomatic osteoporotic compression fractures of the lumbar-sacral area.

This retrospective study was conducted by reviewing the medical records and radiological reports and by reading magnetic resonance (MR) images from August 2013 to July 2016. We identified 1233 cases with symptomatic vertebral compression fractures for which surgical interventions were performed. A total of 1144 cases were eligible for this study. Neglected diagnoses by radiologists and clinical physicians were calculated, respectively.

The MR imaging (MRI) findings of SIFs were divided into the body (S1, S2, S3, and S4 levels) and alar areas (unilateral, bilateral, transverse, and none).

A total of 34 (3.00%) cases with SIFs were identified through MRI. A significant difference was observed between 19 (6.53%) patients aged >80 years and 15 (1.76%) aged <80 years (*P* < .0001). Eight (23.53%) and 26 (76.47%) cases of SIFs were neglected by radiologists and clinical physicians, respectively. The S2 and S3 levels were the predominantly involved area (23/34; 67.65%). Furthermore, the bilateral alar area was the most commonly involved (19/34; 55.88%), as observed in coronal views of MRI.

While treating other levels of osteoporotic compression fractures, radiologists and clinical physicians should be aware of SIFs, particularly when the patients are aged >80 years. The coronal oblique MR images of the thoracolumbar region should be carefully read to avoid neglecting SIFs.

## 1 Introduction

Laurie^[1]^ was the first to describe spontaneous osteoporotic sacral fractures; he reported that the common symptoms of sacral insufficiency fractures (SIFs) included severe low back pain,^[2]^ buttock pain, and referred pain to the lower limbs.^[1,3,4]^ The reported risk factors include pelvic radiation therapy,^[5]^ steroid-induced osteopenia, rheumatoid arthritis, multiple myeloma, Paget disease, renal osteodystrophy, joint arthroplasty, lumbosacral fusion, and hyperparathyroidism.^[4,6–8]^ Osteoporosis,^[9]^ the predominant metabolic bone disorder affecting 25 million people in the United States, is the leading cause of SIFs.^[10]^

The clinical symptoms of SIFs are vague, and some combined clinical diseases may have the same symptoms because SIFs tend to occur in patients aged 60 to 70 years.^[4,8]^ The incidence of radiculopathy mimicking spinal stenosis was reported to be approximately 5% to 6%,^[4]^ and sphincter disturbance was also described.^[11]^ Some studies have reported parasymphyseal discomfort because of the high incidence of concomitant pubic rami fractures.^[12]^ In addition, SIFs are typically missed in X-rays.^[13]^ Thus, SIFs are very easily neglected by clinical physicians.^[1,14,15]^

Elderly women with osteoporosis have a high risk of SIFs.^[16]^ In the United States, SIFs are estimated to affect approximately 2% female patients aged >55 years.^[10]^ Patients who received radiotherapy for malignant tumors may be at a risk of SIFs,^[17]^ with a prevalence of 89% for patients undergoing radiotherapy for cervical cancer.^[18]^ The true incidence of SIFs is unknown but has been reported to be approximately 1% to 5% in at-risk populations.^[3,19,20]^ Only some studies have reported on neglected or delayed diagnosis of SIFs after treating other levels of osteoporotic compression fractures.^[21–23]^ Therefore, we conducted a retrospective study to assess the rate of SIFs in patients with symptomatic osteoporotic compression fractures of the lumbar–sacral region. We calculated the rate of neglected diagnosis by radiologists and clinical physicians.

## 2 Materials and methods

This retrospective study was conducted by reviewing the medical records, radiologist reports, and reading from August 2013 to July 2016. We identified 1233 cases with symptomatic vertebral compression fractures, for which surgical interventions were performed. The surgical techniques included vertebroplasty, kyphoplasty, and vertebral body augmentation with intrabody devices (T-Ba or Spine Jerk). Eighty-nine cases were excluded because of malignancy, infective spondylitis, or data error; thus, 1144 cases were eligible for this study.

We recorded the baseline characteristics of these cases, such as age, sex, body mass index, and bone mineral density (BMD). The surgical level of vertebrae was assessed by reviewing medical records and the follow-up radiography results after surgeries. The SIFs were identified by reading MR images because MRI can detect early changes of sacral insufficiency, and similar to bone scintigraphy, with a reported sensitivity of or approximately 100%.^[24]^ Because clinical symptoms of SIFs are vague, the SIFs were defined as bone marrow edema (low signal intensity at T1 weight images and high signal intensity at T2 weight images) at the sacral area including body and sacral alar area. All of MR images were analyzed by 2 observers (Dr F-CK and Dr L-RY) and interobserver agreement was done.

Neglected diagnoses by radiologists and clinical physicians were identified by reviewing the radiologists’ reports and medical records, respectively. The delayed surgical interventions for the SIFs were identified by reviewing the medical records if the SIFs were not mentioned in the chart in the first treatment course, if MRI revealed SIFs, and subsequently if the SIFs were noticed during follow-up and surgeries were performed.

**Figure 1:**
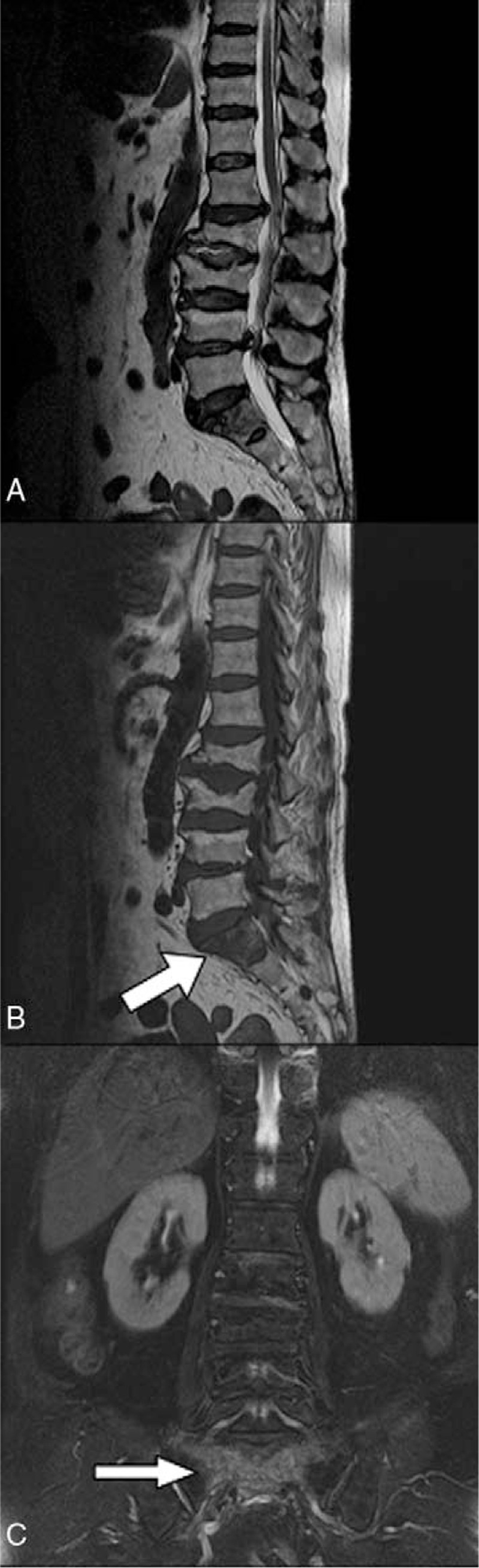
MRI (A, T1-weighted sagittal view; B, T2-weighted sagittal view; and C, T2-weighted coronal view) of an 89-year-old woman with sacral insufficiency fracture. SIF involve S1 body level (white arrow) (B) and (C). MRI = magnetic resonance imaging, SIFs = sacral insufficiency fractures.

**Figure 2:**
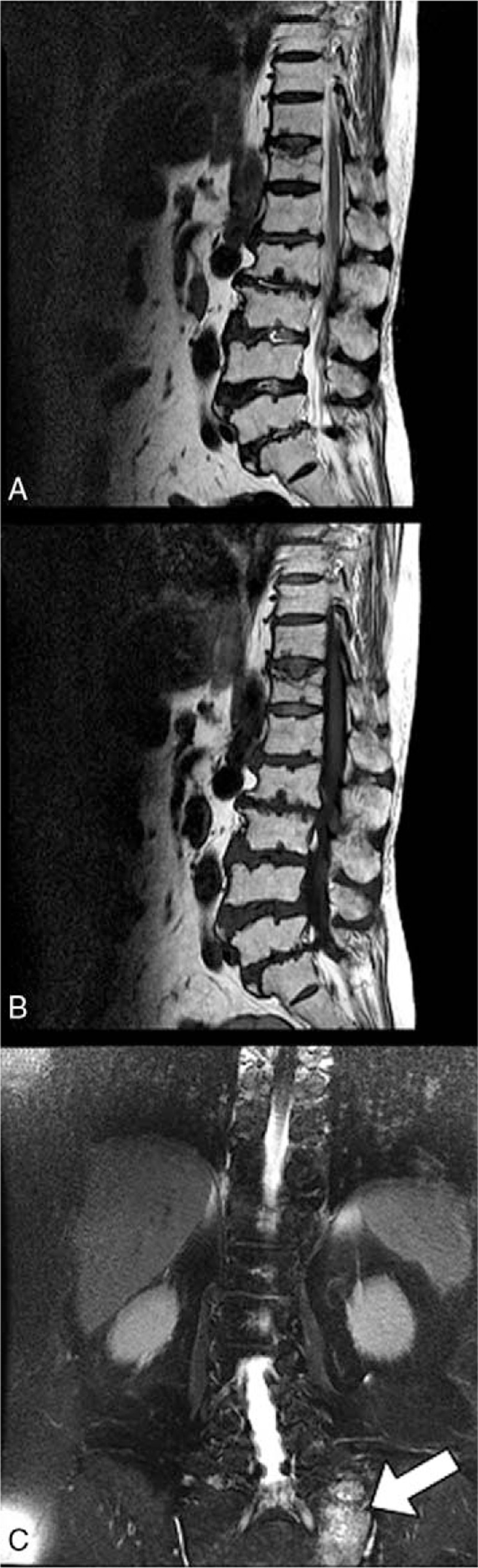
MRI (A, T1-weighted sagittal view; B, T2-weighted sagittal view; and C, T2-weighted coronal view) of an 81-year-old woman with sacral insufficiency fracture. SIF involve left alar area (white arrowheads) (C). MRI = magnetic resonance imaging, SIFs = sacral insufficiency fractures.

**Figure 3:**
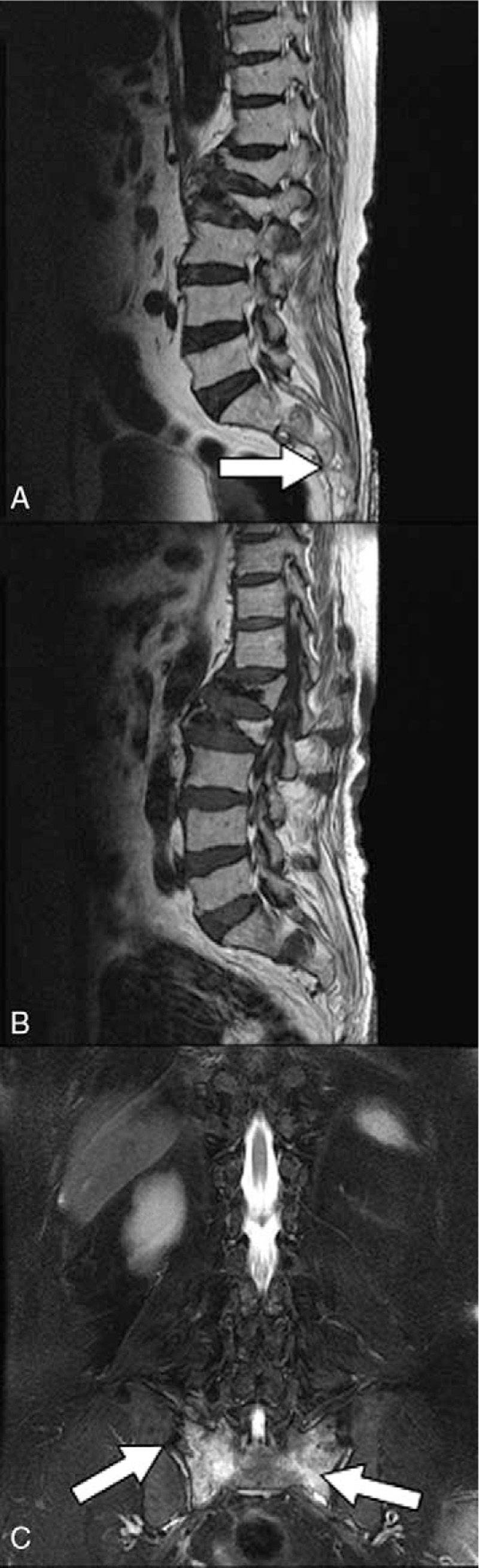
MRI (A, T1-weighted sagittal view; B, T2-weighted sagittal view; and C, T2-weighted coronal view) of an 84-year-old woman with sacral insufficiency fracture. SIF involve S2 body level (white arrow) (A) and bilateral alar area (C). MRI = magnetic resonance imaging, SIFs = sacral insufficiency fractures.

**Figure 4:**
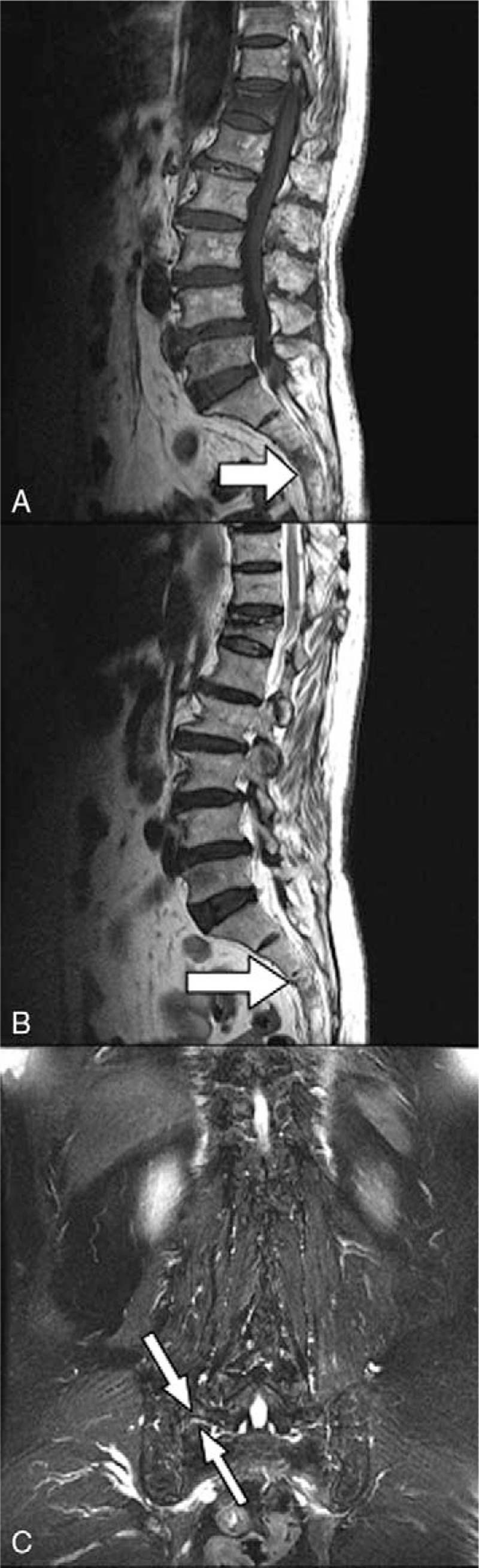
MRI (A, T1-weighted sagittal view; B, T2-weighted sagittal view; and C, T2-weighted coronal view) of a 77-year-old woman with sacral insufficiency fracture. SIF involve S3 body level (white arrow) (B) and transverse alar area (white arrowheads) (C). MRI = magnetic resonance imaging, SIFs = sacral insufficiency fractures.

The MRI findings of the SIFs were divided into body and alar areas (Figs. 1–4). In the body area, the sacral area was divided into S1(sacrum), S2, S3, and S4 levels. In the alar area, the MRI findings were classified as unilateral, bilateral, transverse, and none.

Continuous variables are presented as means and categorical variables as numbers and proportions. The *χ*^2^ test was used to compare the differences between the age groups. The statistical test was considered significant if *P* <.05. The acquisition and analysis of data for this study were approved by the Institutional Review Board (EMRP-106-09).

## 3 Results

Of the 1144 cases with symptomatic vertebral compression fractures, 291 and 853 were aged >80 and <80 years, respectively. A total of 34 (3.00%) cases of SIFs were identified using MR images in this study.

Among the 34 cases with SIFs, 6 were men and 28 were women. Nineteen (6.53%) and 15 (1.76%) cases were aged >80 and <80 years, respectively. The *χ*^2^ test showed significant differences between patients aged >80 or <80 years (*P* <.0001). Thirty (88.24%) cases underwent surgeries for thoracolumbar osteoporotic compression fractures other than the sacral level. The average BMD was −3.04 (−1.1 to −4.8). Sacroplasties were performed in 8 cases: at the first-time admission for 4 cases and months later after surgeries for other vertebral levels for 4 cases. The symptoms of those patients got improvements after sacraoplasties.

Eight (23.53%) and 26 (76.47%) cases of SIFs were neglected by radiologists and clinical physicians, respectively. Ten cases showed persistent severe symptoms during follow-up. Four cases underwent delayed sacroplasty, 1 case received sacro–iliac joint injection, 1 case received spinal fusion from L3 to S1 levels, and 1 case underwent hip arthroplasty. The remaining 3 cases were treated with painkillers alone.

Those cases without sacroplasties had kept painkiller at least 3 months.

The MR images were classified as those of the central body and alar areas (Table 1). One case involved the S1 and S1 levels, and 3 involved the S2 and S3 levels. S2 and S3 levels were predominantly involved (23/34; 67.65%). The bilateral alar was the most commonly involved (19/34; 55.88%), as observed in coronal views of MRI.

**Table 1:**
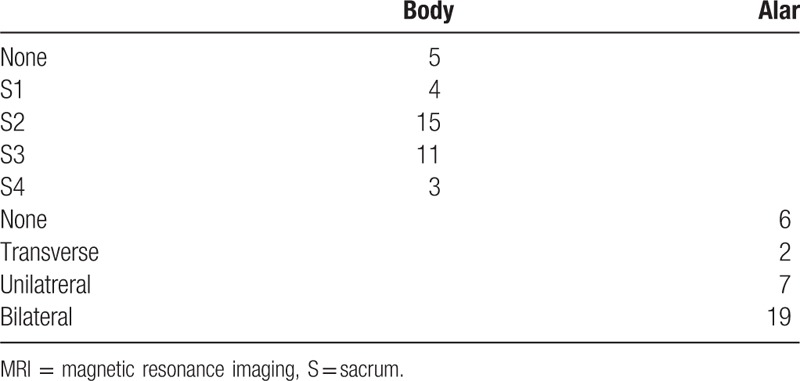
MRI of osteoporotic insufficiency fractures.

## 4 Discussion

SIFs are a type of stress fractures that result from stress applied to osteoporotic bones.^[22]^ The incidence of SIFs has been reported to be 1% to 5%^[3,19]^ in at-risk elderly people. Elderly postmenopausal women with osteoporosis have a higher risk of SIFs^[7]^ than do men. In our study, 82.35% of the SIFs were reported in women. SIFs were reported to frequently occur in patients aged 70 to 75 years.^[25,26]^ In our study, patients aged >80 years had a higher risk of SIFs among patients with symptomatic osteoporotic vertebral compression fractures.

The recommended standard treatment of SIFs is conservative, which includes bed rest, rehabilitation, and analgesics.^[4,26]^ This restricted mobility can persist for a minimum of 3 months or until relief. However, SIFs are typically accompanied by osteopenia or osteoporosis.^[22]^ The average BMD was −3.04 in our study. In the situation, medications, such as antiresorptive agents, for osteoporosis are very crucial for those SIFs cases.^[27]^

SIFs are a common cause of debilitating back pain in the elderly population with a history of trivial fall on the buttocks. Patients typically present with vague low back ache or radiating pain in the buttocks^[28,29]^ but not radiculopathy. The symptoms of SIFs are typically aggravated by axial loading and performing activities; these symptoms generally are vague, mimicking lumbar canal stenosis or metastases. Hence, SIFs are easily missed or underdiagnosed in most circumstances. In addition, radiographies of the lumbosacral spine and pelvis are ordered; however, SIFs are rarely suspected. Studies have reported a delay of approximately 40 to 55 days from symptom onset to sacrum imaging.^[30]^ Only some cases have reported about neglected or delayed diagnosis of SIFs after treating other levels of osteoporotic compression fractures^[14,21–23]^ In our study, 23.53% and 76.47% cases of SIFs were neglected by radiologists and clinical physicians, respectively. Unclear conditions under which SIFs were not identified, some procedures, such as sacroiliac joint injection, hip replacement, or spinal fusion, were performed and might have been ineffective. We recommend that clinical physicians therefore pay more attention to SIFs to make correct diagnosis and select the appropriate treatment strategy.

Bone scintigraphy with technetium Tc 99-labeled methylene diphosphonate may be a sensitive technique for detecting SIFs^[31]^; it has 96% sensitivity and 92% positive predictive rates. Computed tomography can also be used for diagnosis and facilitating the differentiation of SIFs from metastases. The sensitivity rate of computed tomography for SIFs is 60% to 75%.^[24]^ MRI can detect early changes of sacral insufficiency and has a high sensitivity rate of approximately 100%, similar to that of bone scan.^[24]^ T2-short inversion recovery sequences are sensitive in detecting early marrow edema related to SIFs,^[32]^ as early as 18 days after symptom development.^[30]^ The marrow edema of SIFs is demonstrated as areas of high-signal intensity on T2-weighted and inversion-recovery images and low-signal intensity on T1-weighted images.^[24,30,33]^

In case of clinical suspicion of SIFs, coronal oblique images in the plane of the sacrum can demonstrate the vertically oriented fractures.^[32]^ Unfortunately, most patients evaluated for back pain initially did not routinely undergo coronal oblique imaging of the sacrum.^[34]^ Radiologists should be aware of this drawback when interpreting thoracolumbar MR images of elderly patients, particularly in case of concomitant other levels of osteoportic vertebral compression fractures.

The Honda or “H” sign in bone scan was used to diagnose SIFs.^[35]^ However, SIFs do not always symmetrically involve bilateral alar areas, possibly accounting for different target sites while performing sacroplasty in SIFs.^[36]^ In this study, we described the methods of reading MR images for SIFs. We divided the sacral areas in MR images into sacral body and alar areas. We observed that the S2 or S3 levels were predominantly involved areas (67.65%), and bilateral alar was the most commonly involved (55.88%), as observed in coronal views of MRI. We believe that this method to read MR images for SIFs is beneficial when considering target areas for sacroplasty to treat painful SIFs.

Our study had some limitations. This retrospective study only evaluated the rate of neglected SIF diagnosis. We did not evaluate whether sacroplasty should be performed simultaneously with vertebropalsty or kyphoplasty for other levels of osteoporotic vertebral compression fractures. Our study population was recruited from only 1 hospital and had symptomatic vertebral compression fractures. We do not know the exact incidence of SIFs in the general population.

In conclusion, when radiologists and clinical physicians treat other levels of osteoporotic compression fractures, they should be aware of SIFs, particularly if the patients are aged >80 years. The coronal oblique MR images of the thoraco–lumbar region should be carefully read to avoid neglecting SIFs.

## Acknowledgment

The authors thank Ms Chen Tzu-Shan for her efficient assistance.
